# Chemoresistance to gemcitabine in hepatoma cells induces epithelial-mesenchymal transition and involves activation of PDGF-D pathway

**DOI:** 10.18632/oncotarget.1471

**Published:** 2013-10-07

**Authors:** Qiong Wu, Rui Wang, Qingling Yang, Xin Hou, Sulian Chen, Yueyue Hou, Changjie Chen, Yan Yang, Lucio Miele, Fazlul H Sarkar, Yuqing Chen, Zhiwei Wang

**Affiliations:** ^1^ Department of Medical Oncology, First Affiliated Hospital of Bengbu Medical College, Bengbu, Anhui, China; ^2^ Department of Biochemistry and Molecular Biology, Bengbu Medical College, Anhui, China; ^3^ Department of Respiration, First Affiliated Hospital of Bengbu Medical College, Bengbu, Anhui, China; ^4^ University of Mississippi Cancer Institute, Jackson, MS, USA; ^5^ Department of Pathology and Oncology, Karmanos Cancer Institute, Wayne State University, Detroit, MI; ^6^ Department of Pathology, Beth Israel Deaconess Medical Center, Harvard Medical School, MA, USA; ^7^ The Cyrus Tang Hematology Center, Jiangsu Institute of Hematology, the First Affiliated Hospital, Soochow University, Suzhou, China

**Keywords:** Hepatocellular carcinoma, chemoresistance, PDGF-D, EMT, gemcitabine

## Abstract

Hepatocellular carcinoma (HCC) is one of the common malignances in the world and has high mortality in part due to development of acquired drug resistance. Therefore, it is urgent to investigate the molecular mechanism of drug resistance in HCC. To explore the underlying mechanism of drug resistance in HCC, we developed gemcitabine-resistant (GR) HCC cells. We used multiple methods to achieve our goal including RT-PCR, Western blotting analysis, transfection, Wound-healing assay, migration and invasion assay. We observed that gemcitabine-resistant cells acquired epithelial-mesenchymal transition (EMT) phenotype. Moreover, we found that PDGF-D is highly expressed in GR cells. Furthermore, down-regulation of PDGF-D in GR cells led to partial reversal of the EMT phenotype. Our findings demonstrated that targeting PDGF-D could be a novel strategy to overcome gemcitabine resistance in HCC.

## INTRODUCTION

Hepatocellular carcinoma (HCC) is a highly aggressive malignant disease, which predicted 26,190 newly diagnosed cases and 19,590 deaths in the United States in 2012 [1]. Approximately 70% of the patients diagnosed with HCC cannot have curative surgery due to metastasis at the time of diagnosis, resulting in the median overall survival of only few months [1]. The high mortality is also partly due to acquired drug resistance during chemotherapy treatment [2]. It is well known that chemotherapy is a critical management for advanced HCC [3]. Thus, it is pivotal to explore the underlying mechanism of drug resistance and subsequently find ways to overcome such drug resistance for achieving better treatment outcome in HCC patients.

Gemcitabine (2',2'-difluorodeoxycytidine), a deoxycytidine analogue, is used as a chemotherapeutic drug or its combination with other agents for the treatment of advanced HCC [4]. For example, gemcitabine and oxaliplatin (GEMOX) have been considered as second-line treatment in patients with HCC pre-treated with sorafenib [5]. Moreover, chemotherapy with GEMOX prolonged a compete response in advanced fibrolamellar HCC [5]. Recent studies have identified that GEMOX are effective with manageable toxicity in patients with advanced HCC [6]. Additionally, gemcitabine in combination with cisplatin prolonged the survival in advanced HCC [7]. Although gemcitabine is a promising drug to be considered for the treatment of HCC, tumor cells acquire resistance to gemcitabine that causes treatment failure for improving the survival of HCC patients. Thus, it is necessary to understand the underlying mechanism of drug resistance to gemcitabine and find a novel therapeutic strategy for effective treatment of patients with advanced HCC.

Mounting evidence demonstrates that chemo-resistance is associated with the acquisition of epithelial-mesenchymal transition (EMT)-like phenotypic change of cancer cells [8]. EMT is a process by which epithelial cells switch to mesenchymal phenotypic cells, resulting in increased motility and invasion [9]. During EMT, cells lose epithelial cell-cell junction and epithelial markers such as E-cadherin, as well as gain mesenchymal properties with high expression of mesenchyaml molecular markers including Vimentin, Snail, Slug, zinc-finger E-box binding homeobox 1 (ZEB1) and ZEB2 [10]. Multiple studies have revealed that chemo-resistance cells often acquired EMT phenotype [8]. Our previous studies have shown that gemcitabine-resistant (GR) pancreatic cancer cells showed phenotypic changes consistent with EMT and up-regulated Notch pathway [11]. In the current study, we found that GR HCC cells acquired EMT characteristics. Moreover, we identified that Platelet-derived growth factor-D (PDGF-D) signaling pathway is involved in the acquisition of EMT phenotype of GR HCC cells. Furthermore, inhibition of PDGF-D pathway partly reversed EMT to mesenchymal-epithelial transition (MET). Our findings suggest that PDGF-D pathway is involved in chemo-resistance and EMT characteristics of HCC cells, demonstrating that targeting PDGF-D could overcome resistance of HCC to gemcitabine. Therefore, inhibition of PDGF-D may have therapeutic implications for the successful treatment of HCC patients.

## RESULTS

### Establishment of gemcitabine-resistant HCC cell lines

To develop HCC cell lines chronically resistant to gemcitabine, HepG2 and SMMC-7721 cells were exposed to increasing concentrations of gemcitabine. Specifically, to establish gemcitabine resistant HCC cell line, cells were continuously exposed to gemcitabine for more than 12 months. After surviving cells reached more than 70% confluency, they were passaged by trypsinization and exposed to increased concentration of gemcitabine. The process was repeated until resultant HCC cells displayed resistance to the growth inhibitory properties of 10 μg/ml gemcitabine. The resulting cells were designated as HCC GR cells. HCC GR cells were cultured for additional 3 months in DMEM medium containing 10 μg/ml gemcitabine for this study.

### Morphologic changes in HCC GR cells

**Figure 1:**
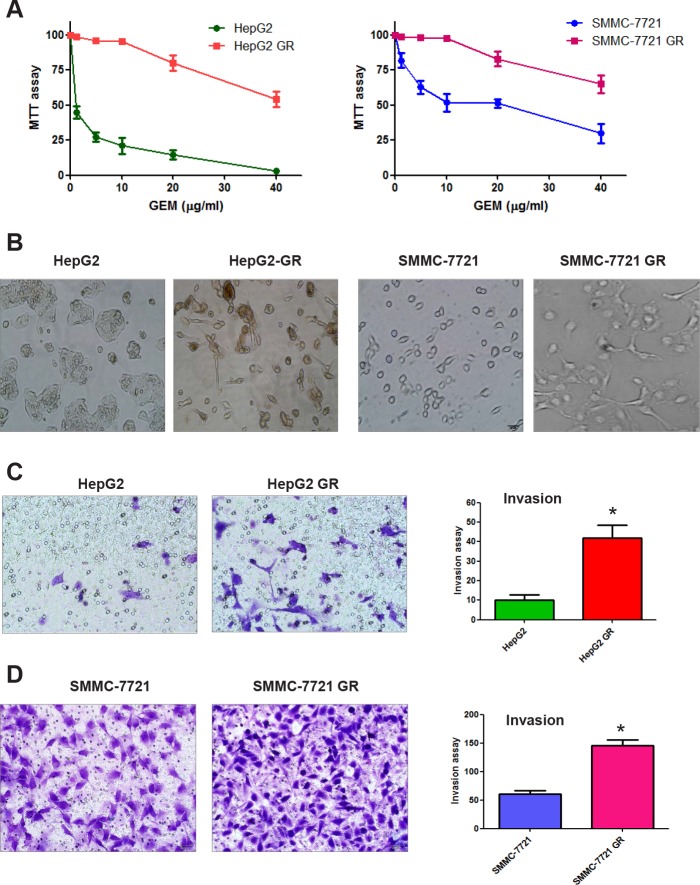
HCC gemcitabine-resistant (GR) cells acquired EMT phenotype A, MTT assay was performed in parental HepG2, SMMC-7721, HepG2 GR, and SMMC-7721 GR cells, respectively. B, Cell morphology was observed by microscopy. Parental HepG2 and SMMC-7721 cells displayed an epithelioid and cobblestone appearance with little pseudopodia. In contrast, HepG2 GR and SMMC-7721 GR cells showed loss of cell polarity and increased formation of pseudopodia, leading to elongated, irregular fibroblastoid morphology. C, Invasion assay was conducted to measure the invasive capacity in HepG2 and HepG2 GR cells. * P<0.05 vs control. D, Invasion assay was performed to detect the invasive activity in SMMC-7721 and SMMC-7721 GR cells. * P<0.05 vs control.

Our previous study has shown that GR pancreatic cancer cells had morphologic changes consistent with EMT [11]. Therefore, we tested whether HCC GR cells have markedly morphologic changes compared with the parental cell lines. As illustrated in Figure 1B, HepG2 and SMMC-7721 cells displayed a rounded shape and little formation of pseudopodia. In contrast, HepG2 GR and SMMC-7721 GR cells had phenotypic changes including loss of cell polarity and increased formation of pseudopodia, leading to elongated, irregular fibroblastoid morphology (Figure 1B). These morphologic changes suggest that HCC GR cells acquired a mesenchymal phenotype.

### Increased invasion activity in HCC GR cells

It has been known that after EMT, cells enhance migratory and invasive activity. To confirm the EMT progress in HCC GR cells, we conducted the invasion assay. We found that HepG2 GR cells showed approximately 4-fold increase in the number of cells migrating through a Matrigel-coated membrane compared with HepG2 cells (Figure 1C). Consistently, SMMC-7721 GR cells have significantly increased invasion activity (Figure 1D).

### HCC GR cells have increased motility activity

**Figure 2:**
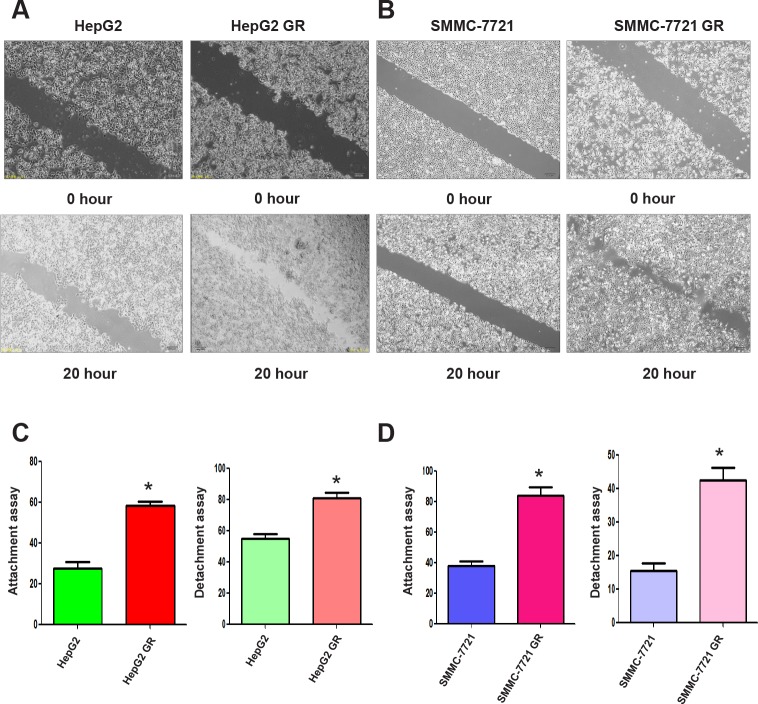
HCC GR cells have increased migratory capacity A, Wound assays were performed to compare the migratory potential of HepG2 and HepG2 GR cells. B, Wound assays were conducted to detect the migratory capacity of SMMC-7721 and SMMC-7721 GR cells. C, Cell attachment and detachment assays were conducted in HepG2 and HepG2 GR cells. * P<0.05 vs control. D, Cell attachment and detachment assays were performed in SMMC-7721 and SMMC-7721 GR cells. * P<0.05 vs control.

To further confirm whether HCC GR cells acquired EMT characteristics, we compared the migratory potential of HCC GR cells and parental HCC cells using a scratch wound-healing assay. As demonstrated in Figure 2A, 2B, HepG2 GR and SMMC-7721 GR cells have significantly increased numbers of cells migrating across the wound, suggesting that HCC GR cells acquired enhanced migration capacity.

### HCC GR cells have enhanced detached and attachment activity

It has been well accepted that cell detachment from the matrix is a crucial component in cancer spreading, often leading to tumor recurrence. Cancer cell attachment to the secondary site is the hallmark of tumor metastatic process. Consistent with this notion, we found that HepG2 GR and SMMC-7721 GR cells have increased capacity of attachment and detachment (Figure 2C, 2D).

### HCC GR cells have EMT marker changes

**Figure 3:**
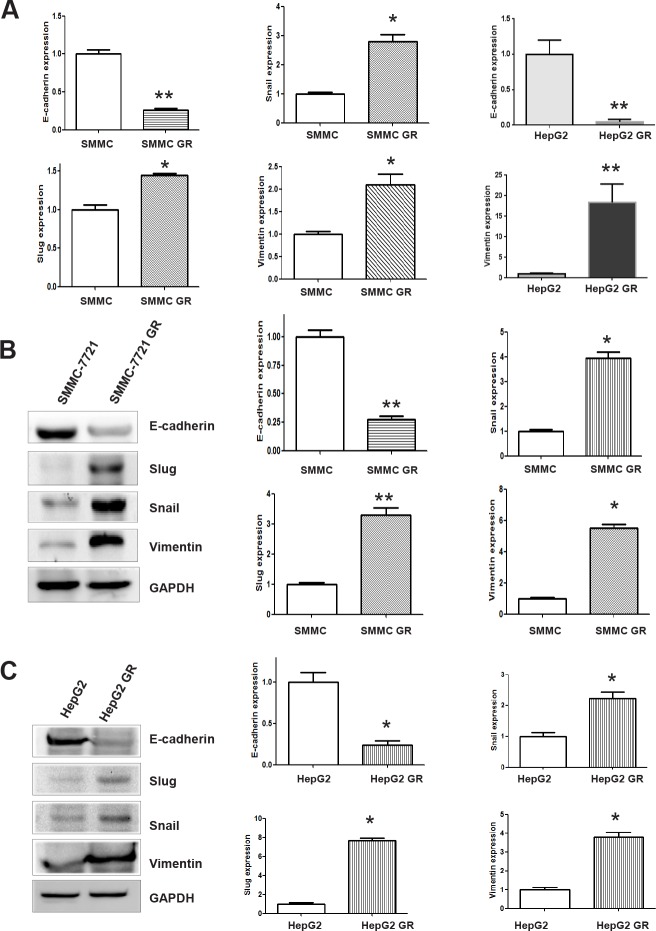
HCC GR cells have high expression of EMT markers A, RT-PCR assay was performed to detect the mRNA levels of E-cadherin, Slug, Snail, and Vimentin in HepG2 GR and SMMC-7721 GR cells. * P<0.05 vs control. B, Left panel, Western blotting analysis was conducted to measure the expression of E-cadherin, Snail, Slug, and Vimentin in SMMC-7721 and SMMC-7721 GR cells. Right panel, Quantitative results are illustrated for left panel. SMMC: SMMC-7721. * P<0.05 vs control. C, Left panel, Western blotting analysis was used to detect the expression of E-cadherin, Snail, Slug, and Vimentin in HepG2 and HepG2 GR cells. Right panel, Quantitative results are illustrated for left panel. SMMC: SMMC-7721. * P<0.05 vs control.

To further identify whether HCC GR cells have the specific molecular changes consistent with EMT, we measured the expression of markers of epithelial and mesenchymal phenotypes using RT-PCR and Western blotting analysis, respectively. We observed that the expression of epithelial adhesion molecule E-cadherin was significantly reduced in HepG2 GR and SMMC-7721 GR cells (Figure 3). On the contrary, the expression of mesenchymal markers including Vimentin, Snail and Slug was elevated in HepG2 GR and SMMC-7721 GR cells (Figure 3), indicating that the expression of these factors plays a critical role in gemcitabine-induced EMT of HCC cell.

### Activation of PDGF-D pathway in HCC GR cells

**Figure 4:**
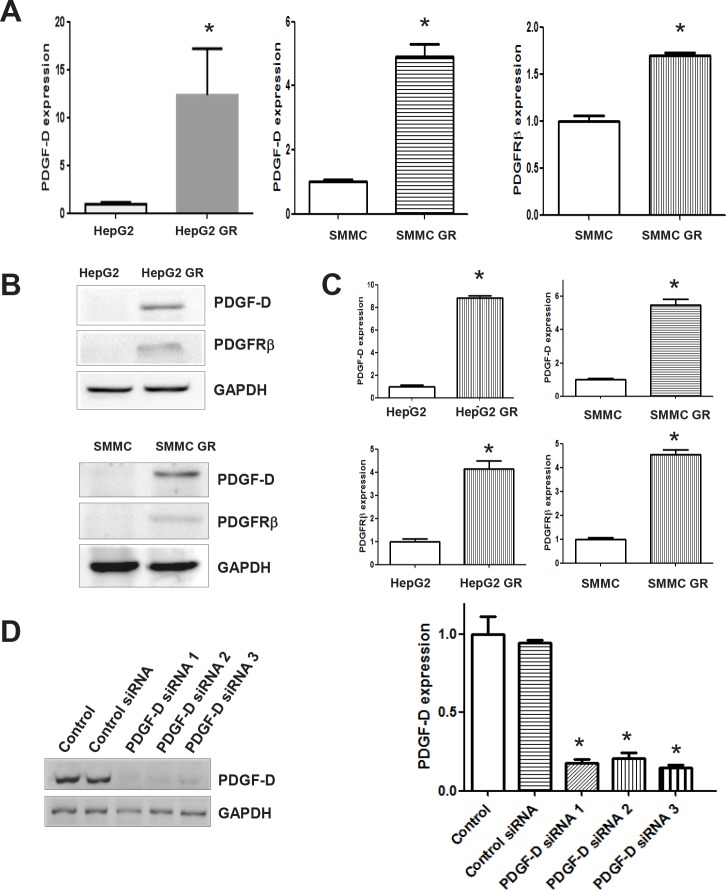
HCC GR cells have high expression of PDGF-D A, RT-PCR assay was done to quantify the mRNA level of PDGF-D in HepG2 GR and SMMC-7721 GR cells. SMMC: SMMC-7721. * P<0.05 vs control. B, Western blotting analysis was performed to detect the expression of PDGF-D and PDGFRβ in HepG2 and HepG2 GR cells. SMMC: SMMC-7721. C, Quantitative results are illustrated for panel B. * P<0.05 vs control. D, Left paenl, Western blotting analysis was conducted to detect the inhibitory efficacy of PDGF-D siRNA in HepG2 GR cells. Right panel, Quantitative results are shown for left panel * P<0.05 vs control.

Since PDGF-D has been reported to play a pivotal role in the EMT induction during tumor progression [12], we measured the expression of PDGF-D at mRNA and protein levels by RT-PCR and western blotting, respectively. We observed an increased activation of PDGF-D at both mRNA and protein levels in HepG2 GR and SMMC-7721 GR cells (Figure 4A, 4B). Moreover, we found that the expression of PDGFRβ was increased in HepG2 GR and SMMC-7721 GR cells compared with their parental cells (Figure 4B). Consistently, we also found the activation of PDGF-D pathway in BxPC-3 GR and PANC-1 GR pancreatic cancer cells (data not shown), suggesting that PDGF-D may be involved in gemcitabine-induced EMT in human cancer cells.

### Down-regulation of PDGF-D reverses EMT to MET in GR cells

**Figure 5:**
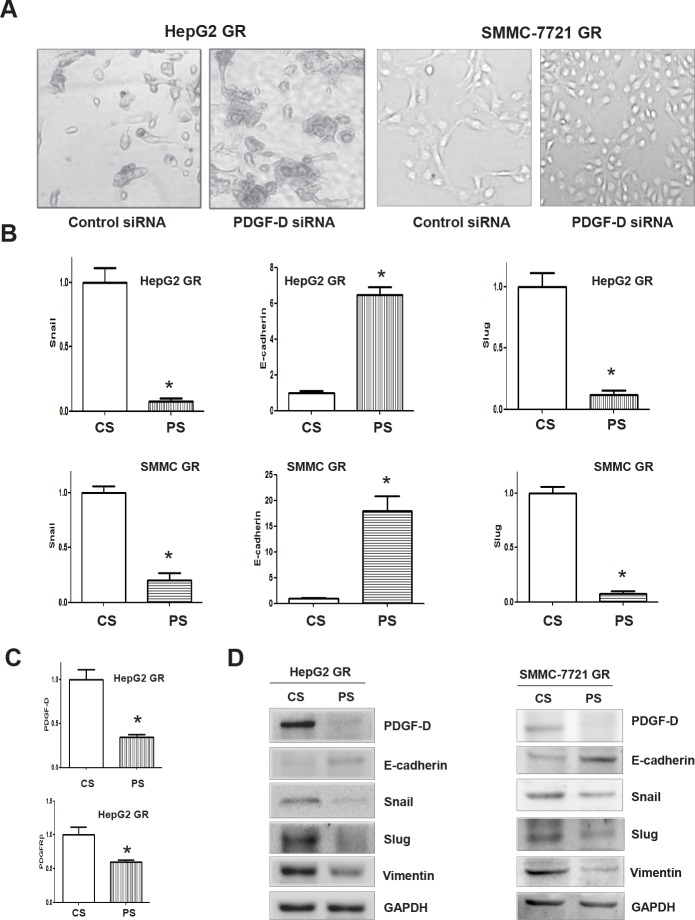
PDGF-D contributes to the regulation of EMT markers in HCC GR cells A, Down-regulation of PDGF-D caused reversal of EMT phenotype of HCC GR cells. HepG2 GR and SMMC-7721 GR cells transfected with control siRNA exhibited a fibroblastic-type phenotype, while these GR cells transfected with PDGF-D siRNA display round-like epithelial cell shape. B, HCC GR cells transfected with control siRNA or PDGF-D siRNA were used for assessing the expression of markers of epithelial and mesenchymal phenotypes using Real-time RT-PCR. SMMC: SMMC-7721. CS: control siRNA; PS: PDGF-D siRNA. * P<0.05 vs control. C, Real-time RT-PCR was used to quantify PDGF-D and PDGFRβ mRNA expression in HepG2 GR cells transfected with PDGF-D siRNA. CS: control siRNA; PS: PDGF-D siRNA. *, P<0.05 compared with control siRNA. D, HCC GR cells transfected with control siRNA or PDGF-D siRNA were used for assessing the expression of markers of epithelial and mesenchymal phenotypes by Western blotting analysis. CS: control siRNA; PS: PDGF-D siRNA.

To further confirm the role of PDGF-D in HCC GR cells containing EMT features, we explored whether inhibition of PDGF-D by its specific siRNA could reverse EMT to MET. We found that PDGF-D siRNAs remarkably inhibited the expression of PDGF-D in HepG2 GR cells (Figure 4D) and SMC-7721 GR cells (data not shown). Subsequently, we assessed whether EMT phenotype was reversed in HCC GR cells transfected with PDGF-D siRNA. We observed that HepG2 GR and SMMC-7721 GR cells transfected with PDGF-D siRNA displayed round cell-like morphology (Figure 5A). Moreover, we found that the expression of E-cadherin was significantly increased, while the expression of mesenchymal markers was decreased in HCC GR cells transfected with PDGF-D siRNA (Figure 5B, 5D), suggesting that down-regulation of PDGF-D led to the reversal of EMT to MET phenotype.

### Down-regulation of PDGF-D signaling enhances detachment and inhibits the migration and invasion of HCC GR cells

**Figure 6:**
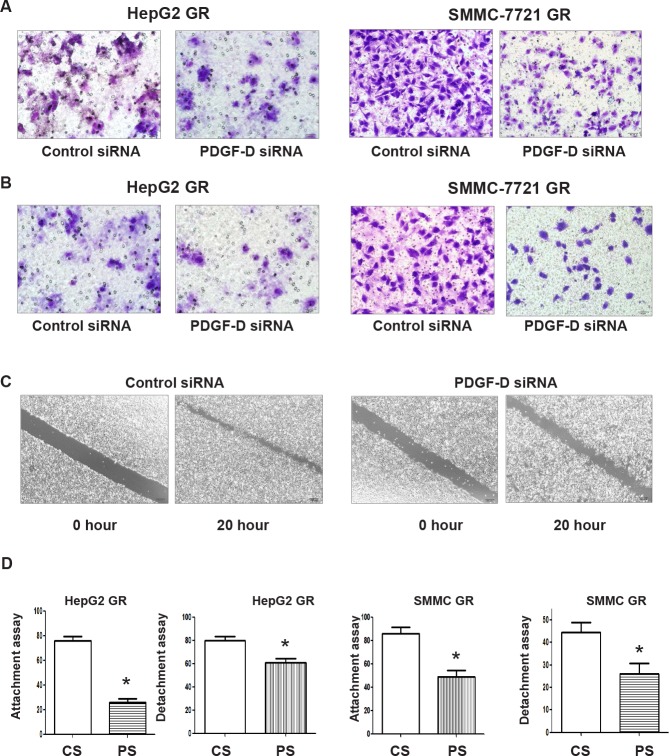
Down-regulation of PDGF-D inhibited cell migration and invasion, and reduce detachment of HepG2 GR cells A, Transfection of HepG2 GR cells and SMMC-7721 GR cells with PDGF-D siRNA inhibited cell migration. B, PDGF-D siRNA suppressed cell invasion of HepG2 GR and SMMC-7721 GR cells by Transwell invasion assay. C, SMMC-7721 GR cells transfected with PDGF-D siRNA caused decreased motility capacity as assessed by Wound healing assay. D, HepG2 GR and SMMC-7721 GR cells transfected with PDGF-D siRNA inhibited the attachment and detachment of cells. CS: control siRNA; PS: PDGF-D siRNA. * P<0.05 vs control siRNA.

To further validate the reversal of EMT by PDGF-D siRNA, we measured the cell detachment, attachment, and motility capacities. As expected, we observed that down-regulation of PDGF-D by siRNA markedly reduced the migratory (Figure 6A) and invasive ability of HCC GR cells (Figure 6B). Consistent with these results, PDGF-D siRNA inhibited cell motility as assessed by wound healing assay in SMMC-7721 GR cells (Figure 6C). Moreover, HCC GR cells transfected with PDGF-D siRNA displayed decreased detachment and attachment capacity (Figure 6D). These results clearly suggest that down-regulation of PDGF-D inhibited PDGFRβ and consequently up-regulated E-cadherin as well as down-regulated mesenchymal protein expressions, resulting in the reversal of the EMT to a MET phenotype with less cell migration and invasion characteristics.

## DISCUSSION

HCC is the one of leading causes of cancer-related deaths in the United States [1]. Surgically unresectable patients have a poor prognosis due to distant metastases and in part due to intrinsic and acquired resistance to chemotherapeutic drugs. A line of evidence has revealed that drug resistance can be divided into oncogenic and nononcogenic [13, 14]. Nononcogenic mechanisms include expression of energy-dependent drug transporters that eject anti-cancer drugs from cells, induction of drug detoxification, and mutation in drug-targets [15]. Oncogenic drug resistance is due to activation of cellular pathways involved in cell proliferation and survival, leading to uncontrolled malignant growth [13]. In line with this concept, multiple key cellular signaling pathways, including Akt, mTOR (mammalian target of rapamycin), NF-κB (nuclear factor-kappa B) and Notch, have been demonstrated to be involved in drug resistance to conventional chemotherapeutics [16-20]. Although the causes of drug resistance have been explored for many years, the mechanisms responsible for drug resistance are still largely elusive [21, 22]. Therefore, elucidation of the underlying mechanism of drug resistance is important to develop novel strategies for effective treatment of advanced HCC patients.

Recently, accumulating evidence has demonstrated that drug-resistant cancer cells are associated with the EMT process in human cancers including HCC. For example, Tamoxifen-resistant MCF7 breast cancer cells showed EMT characteristics with altered β-catenin phosphorylation [23]. Similarly, oxaliplatin-resistant colorectal cancer cells underwent EMT progression [24]. Paclitaxel-resistant ovarian cancer cells displayed decreased E-cadherin expression, and increased expression of mesenchymal markers consistent with EMT phenotype [25]. Moreover, gefitinib-resistant lung cancer cells have EMT phenotype with down-regulation of E-cadherin and up-regulation of Vimentin [26]. In line with these reports, we have previously observed that gemcitabine-resistant pancreatic cancer cells acquired EMT features [11]. Consistent with these findings, in the current study, we found that HepG2 GR cells and SMMC-7721 GR cells demonstrated altered morphological characteristics of cells similar to EMT with decreased E-cadherin and increased Vimentin, Snail and Slug, suggesting that there is a link between chemo-resistance and EMT in HCC.

PDGF-D signaling pathway has been reported to be involved in the regulation of various cellular processes, such as cell proliferation, apoptosis, invasion, metastases and EMT in human cancer [27]. It is known that PDGF-D exerts its biological function through specifically binding to and activating its cognate receptor PDGFR-β, leading to phosphorylation of PDGFR-β and subsequent activation of its target genes such as PI3K/Akt, mTOR, Notch, NF-κB, CXCR4 (C-X-C chemokine receptor type 4), and Bcl-2 [28, 29]. Our previous studies have shown that PDGF-D could facilitate EMT in prostate cancer cells [30, 31]. Specifically, over-expression of PDGF-D caused EMT phenotype in PC3 prostate cancer cells with loss or relocation of E-cadherin and increased expression of Vimentin and Nestin, suggesting that PDGF-D overexpression contributes to EMT in human cancers [31]. Moreover, miR-200 regulated PDGF-D-mediated EMT partly through down-regulation of ZEB1, ZEB2 and Snail, and up-regulation of E-cadherin in prostate cancer cells [32]. In agreement with the role of PDGF-D in EMT, we observed high expression of PDGF-D in HCC GR cells consistent with EMT phenotype. More importantly, down-regulation of PDGF-D in HepG2 GR and SMMC-7721 GR cells reversed EMT to MET, demonstrating that PDGF-D plays an important role in GR-induced EMT in HCC.

**Figure 7:**
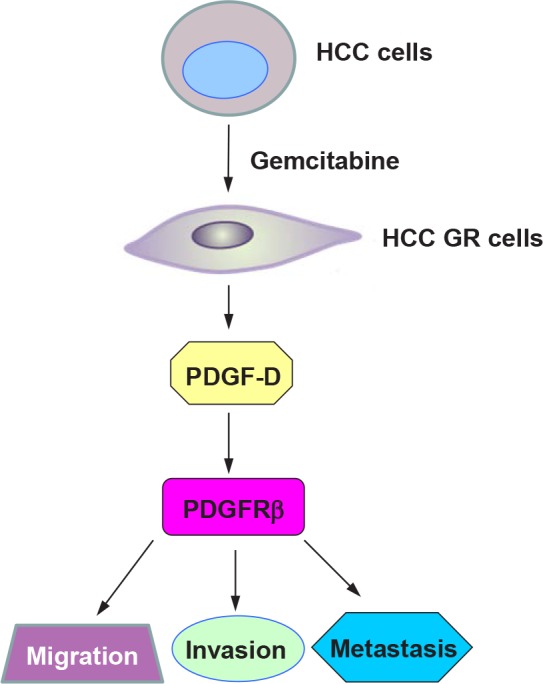
A proposed model for PDGF-D signaling pathway in HCC GR EMT-type cells HCC GR cells acquired EMT phenotype where PDGF-D was up-regulated, leading to enhanced migration, invasion, and metastasis.

A number of studies have demonstrated that cells that acquired drug resistant via nononcogenic do not become increasing malignant [15]. However, resistance to a drug through activation of oncogenic pathways is associated with highly aggressive cancer phenotype including enhanced invasiveness, metastasis, and poor overall survival [13]. In support of this note, we found that PDGF-D was highly expressed in HCC GR cells, which is consistent with its function in the acquisition of EMT phenotype and enhanced migration and invasion (Figure 7). More importantly, inhibition of PDGF-D led to the reversal of EMT to MET, resulting in decreased invasive behavior of HCC GR cells. These results suggest that the inactivation of PDGF-D could be a promising approach for overcoming chemoresistance toward effective treatment outcome of advanced HCC patients.

## MATERIALS AND METHODS

### Cell culture, reagents and antibodies

HepG2 and SMMC-7721 cells were cultured at 37°C in 5% CO_2_ in Dulbecco's modified Eagle's medium (DMEM; Gibco, Gaithersburg, MD, USA) supplemented with 10% fetal bovine serum. MTT [3-(4,5-dimethythiazol-2-yl)-2,5-diphenyl tetrazolium bromide] was purchased from Sigma (St. Louis, Mo). Primary antibodies against E-cadherin, Snail, Slug, Vimentin, GAPDH, PDGF-D, and PDGFRβ were bought from Santa Cruz Biotechnology (Santa Cruz, CA). The secondary antibodies were also obtained from Santa Cruz Biotechnology.

### Cell proliferation studies by MTT assay

The HCC cells and HCC gemcitabine-resistant (GR) cells (5 ×10^3^) were seeded at equal densities into a 96-well culture plate for overnight incubation. Then, the medium was replaced with medium containing different concentrations of gemcitabine for 72 hours. MTT assay was conducted as described before [33].

### Wound healing assay

The HCC cells and HCC GR cells were seeded in 6-well plate until the cells grew to 90–95% confluency. The scratch wound was generated in the surface of the plates using a pipette tip. Photographic images were taken from HCC and HCC GR cells at 0 hour and 16 hours.

### Cell attachment and detachment assay

Cell attachment and detachment assays were conducted as described before [31]. Briefly, for attachment assay, HCC cells, HCC GR cells, and GR cells transfected with PDGF-D siRNA were seeded in 24-well plates at 5 × 10^4^ cells per well. Unattached cells were removed after 1 hour incubation, and the attached cells were counted after trypsinization. The data were presented as a percentage of the attached cells compared to total cells. For cell detachment assay, after 24 hours incubation, the cells were incubated with 0.05% trypsin for 3 minutes to detach the cells. Then, the culture medium was added to inactivate the trypsin and the detached cells were collected. The remaining cells were incubated with 0.25% trypsin to detach and counted. The data were presented as a percentage of the detached cells to total cells.

### Transwell migration and invasion assays

The migration of HCC cells was conducted using a 24-well Transwell chamber (Corning) with gelatin-coated polycarbonate membrane filter. The invasive capacity of HCC cells was performed using Transwell inserts with Matrigel (BD Biosciences). After incubation for 16 h, the upper surfaces of the Transwell chambers were scraped with cotton swabs, and the migrated and invaded cells were fixed with 4% paraformaldehyde, and then stained with Giemsa solution. The stained cells were photographed and counted under a light microscope in five randomly-selected fields.

### RNA extraction and reverse transcription-PCR analysis for gene expression

The total RNA from HCC cells and HCC GR cells was isolated with Trizol (Invitrogen) and purified with RNeasy Mini Kit and RNase-free DNase Set (Qiagen) according to the manufacturer's protocols. The primers used in the PCR reactions are listed in Table I. The expression of GAPDH was used as internal control. RT-PCR amplifications were performed as described before [11, 34].

**Table 1:** The primers used for RT-PCR analysis

Gene	Prime 5'to 3'
E-cadherin_F1	GAAGTGTCCGAGGACTTTGG
E-cadherin_R1	CAGTGTCTCTCCAAATCCGATA
Vimentin_F1	TGTCCAAATCGATGTGGATGTTTC
Vimentin_R1	TTGTACCATTCTTCTGCCTCCTG
PDGF-D_F1	CCCAGGAATTACTCGGTCAA
PDGF-D_R1	ACAGCCACAATTTCCTCCAC
GAPDH_F1	CAGCCTCAAGATCATCAGCA
GAPDH_R1	TGTGGTCATGAGTCCTTCCA

### Protein extraction and Western blotting

Cells were harvested and lysed with RIPA buffer (1 × PBS, 1% Nonidet P40, 0.5% sodium deoxycholate, 0.1% SDS, and protease inhibitor cocktail). The protein concentrations were measured using the Bio-Rad protein assay kit (Bio-Rad Laboratories, CA). Immunoblotting was conducted with standard protocols as described previously [35].

### Transfection

Cells were seeded in six-well plates and transfected with PDGF-D siRNA, control siRNA using Lipofectamine 2000 as described earlier [36]. The sequences for PDGF-D siRNA and control siRNA are listed in Table II. After the indicated periods of incubation, the cells were subjected to further analysis as presented under the results section.

**Table 2:** The RNA sequences for specific siRNAs

Gene	Prime 5' to 3'
PDGF-D_F1	GGAUACAGCUAGUGUUUGATT
PDGF-D_R1	UCAAACACUAGCUGUAUCCTT
PDGF-D_F2	GGCAAGAAGAUCUUGAGAATT
PDGF-D_R2	UUCUCAAGAUCUUCUUGCCTT
PDGF-D_F3	CCAGGAAUUACUCGGUCAATT
PDGF-D_R3	UUGACCGAGUAAUUCCUGGTT
Control_F1	UUCUCCGAACGUGUCACGUTT
Control_R1	ACGUGACACGUUCGGAGAATT

### Statistical Analysis

Values were shown as means± SEM and analyzed using GraphPad Prism 4.0 (Graph pad Software, La Jolla, CA). Statistical comparisons between different groups were performed using Student *t* test. P<0.05 was considered statistically significant.
